# Can a Person Be Anæsthetized during Sleep?

**Published:** 1874-05

**Authors:** 


*Can a Person be Anæsthetized During Sleep?*—The Medical journals are discussing this very important medico-legal question. We take the following from the *Medical and Surgical Reporter and the Pacific Journal:*

"This question is ably discussed, though not conclusively settled, by Professor Dolbeau, in the January number of the *Annales d' Hygiene*. The case was that of a young woman who claimed she had been chloroformed during sleep and then violated. Professor Dolbeau performed several experiments, and found that sleeping animals were readily aroused by the presence of even small quantities of chloroform in their immediate vicinity. The cases of three patients are also given, who while sleeping were readily aroused by applying small quantities of chloroform at no great distance from the nostrils. In a second series of experiments made on seven patients, ten drops of chloroform were poured on a napkin folded in four, which was gradually brought to the vicinity of the air-passages, so that all air inspired had traversed it. In all these cases the patients were suddenly aroused from their sleep, some immediately, and one only after the eleventh inspiration.

A third group of cases, consisting of twenty-nine patients, was next experimented upon, furnishing different results. These are given in some detail, but it will suffice to say that it was found that in ten out of the number—that is, in more than a third, complete anæsthesia could be induced without awakening them. Dexterity in the mode of procedure seemed to have something to do with the proportion thus obtained, for this increased progressively with the number of cases experimented upon.

New researches will still be required in order to establish the influence which may be excited on the results by the age of the subjects, their sex, their prior condition of health, personal habits, etc. The purity of the chloroform employed is also a matter of importance. While thus appealing to future researches your reporter, making certain reserves, still feels that he is authorized in drawing a somewhat positive conclusion. Scientifically it is difficult, but often possible, to render persons insensible by means of chloroform who are in a state of natural sleep. Certain precautions, the employment of a very pure article, and great practice, are conditions that favor the success of the attempt. It is probable that certain subjects are absolutely refractory—that is, it is impossible to anæsthetize them, in spite of every precaution that can be taken. Others, on the contrary, and especially young children, easily undergo anæsthesia without being aroused from their sleep by the irritation which the anæsthetic produces in the air passages. Under the criminal aspect it is certain that chloroform administered to sleeping persons may facilitate the perpetration of certain crimes. It is, however, probable that the conditions favorable for anæsthesia will be rarely combined on the occasion of criminal attempt. But before the tribunals the expert should declare that it is possible if not easy, to render a sleeping person sufficiently insensible by chloroform to allow of his becoming the victim of a criminal attempt."

---

